# Why is it so hard for academic medical centers to succeed in value-based care?

**DOI:** 10.1093/haschl/qxad002

**Published:** 2023-06-20

**Authors:** Bob Kocher, Robert M Wachter

**Affiliations:** Department of Health Policy, Stanford University, 3340 Hillview Avenue, Palo Alto, CA 94304, United States; Department of Medicine, University of California, San Francisco, 505 Parnassus Avenue, San Francisco, CA 94117, United States

**Keywords:** academic medical centers, value-based care

## Abstract

Academic medical centers (AMCs) excel in many ways, but struggle to succeed at delivering cost-effective care in value-based payment models. To the extent these payment models become more widespread, or mandatory, this could be a giant risk to the future success of AMCs. Many attributes of AMCs that have served them well in a fee-for-service payment system may hinder the transformation needed to succeed in value-based care. Much of the underperformance of AMCs may be explained by two core competencies that AMCs lack: the limited ability to redesign clinical workflows and inability to change their economic relationships with their own specialists and primary care providers. These limitations, in turn, flow from a combination of electronic medical record systems that lock-in existing practice patterns, compensation systems that reward volume over value, organizational structures that make it very hard to drive clinicians to change, workforces with too many specialists, and complex accounting systems. To preserve current margins in value-based care, AMCs need to reduce their cost structures and gain new skills in primary and preventive care. Alternative, AMCs may choose to eschew value-based care and raise their prices for fee-for-service to offset declines in patient volume.

The U.S. health system is renowned for producing some of the world's greatest academic medical centers (AMCs). Massive institutions like the Mayo Clinic, Johns Hopkins Hospital, and Massachusetts General Hospital perennially dominate “best hospitals” lists and attract patients from all over the world. These and similar systems also excel in training the next generation of clinicians and producing groundbreaking research.

Despite—or perhaps because of—these accomplishments, AMCs have struggled to adapt to the new world of value-based care.^1^ In this commentary, we argue that many of the attributes of AMCs, attributes that have served them well in a fee-for-service payment environment, now hinder the transformation needed to provide high-value care at lower costs. While the failure of transformation has many underlying reasons, we believe that much of it boils down to core competencies that AMCs lack: the limited ability to redesign clinical workflows and inability to change their economic relationships with their own specialists and primary care providers. These limitations, in turn, flow from a combination of electronic medical record systems that tend to calcify existing practice patterns, compensation systems that reward volume over value, organizational structures that make it very hard to motivate or require clinicians to change, workforces with too many specialists, and complex accounting systems redirecting surplus funds to support money-losing but mission-critical education and research programs.

AMCs have been demonstrated to deliver higher quality care at higher cost than non-AMCs.^2^ They achieve these results by offering specialized care across all levels of acuity, delivered by outsized numbers of highly paid specialist clinicians. The combination of large specialist practices, quality outcomes that patients value, and often high market share has resulted in AMCs achieving enviable levels of market power, and robust balance sheets, largely by negotiating effectively for ever-higher fee-for-service (FFS) commercial insurance prices. This position, quite naturally, reduces pressures to improve productivity and supports costly operating models; such pressures are often needed to create the burning platform for significant practice redesign and cost cutting. Moreover, their strong market position generally forces health plans to include AMCs in their networks, which creates confidence among AMC leaders that patient demand will forever remain stable and secure.

These advantages align perfectly with the incentives produced by FFS payment models, in which more activity and higher acuity translate into more financial success (as well as higher rankings and better brand recognition). High reimbursement, often 50%–100% more than Medicare pays, makes commercially insured patients far more profitable than other patients and creates powerful incentives to maximize revenue from this segment.

It's hard to blame AMCs for taking advantage of their market position to maximize profits, partly in the service of supporting their missions. But, when it comes to transforming into high-value and lower total cost of care delivery systems, these incumbent advantages of AMCs may be their Achilles heel. The focus on commercially insured patients and specialty care leads to a lesser focus on primary care, behavioral health, chronic disease management, and virtual care, all of which generate less revenue and lower margins than higher acuity care. Moreover, high-quality care in these areas (by design) can drive down demand for surgery and hospitalizations, which lowers revenue for AMCs. Simply put, for AMCs to grow services that lower the total cost of care runs counter to their economic incentives. And these are precisely the capabilities needed to succeed in value-based care.

Since the 1990s, AMCs have been told that their failure to become expert players in value-based care would lead to their demise, or at best a shrinking role in the healthcare marketplace. But AMCs have been hearing that value-based care is “just around the corner” for more than two decades. Despite the predictions, today value-based payments remain a relatively small portion of their overall reimbursement. This disconnect between the “inevitability” of value-based care and the marketplace reality has created some well-founded skepticism on the part of AMCs that the current clarion call for transformation must be heeded this time.

In fact, the disappointing speed of the transition from “volume to value” in the U.S. healthcare system is partly due to many AMCs having the market power to resist value-based care payment models proffered by health plans. To change, AMCs will need to be convinced that new payment models either pose an existential threat or are more profitable than the FFS status quo. It is our belief that new payment models, enabled in part by new technologies, are on the cusp of taking over larger roles in the payment landscape. Over time, such models will threaten the primacy of AMCs, as primary care providers operating within value-based compensation frameworks will increasingly seek out non-AMC specialists and hospitals who can demonstrate their ability to provide high-quality care at a lower cost. When that happens, the system will then redistribute substantial portions of the savings derived from lower utilization of AMCs to payors, risk-bearing providers, and patients.

It would be an error to assume that a clear market signal of the switch to value-based care is all that's needed to transform AMCs. Even in markets in which value-based reimbursement has taken hold, many AMCs have failed to generate savings, partly because they have tended to embrace one-sided models in which there is no penalty for not saving money. Their lack of success in the value-based arena may be partly due to risk-adjustment models that don’t fully account for the complexity of AMC patients, and to lower levels of competence in risk coding than in systems with more experience in value-based care and coding.^3^ In contrast, in non-AMC settings, the movement away from FFS payment models has often led to a lower total cost of care. Non-AMC health systems like Advocate, Summa, and Beaumont are among the top 20 highest performing accountable care organizations (ACOs).^1^ Most successful ACOs are independent physician groups that benefit economically from the savings derived from lower specialist and hospital utilization. The success of non-AMC health systems in non-FFS systems may be partly due to fewer employed specialist physicians needing their salaries funded and the absence of an imperative to cross-subsidize research and educational missions.

AMCs have a uniquely hard time reducing their specialist workforce since many are also needed for research and teaching, activities that may support a portion of their salaries, and some may also be tenured. The other challenge is that the clinical workforce in AMCs is often composed of faculty who spend significant fractions of their time engaged in teaching and research. Working with part-time clinicians—whether a one- to two-session per week ambulatory provider or a one-month per year inpatient consultant—makes changes in workflow more difficult to plan, operationalize, and communicate.

Another challenge that AMCs face in developing value-based care models is that, in such models, historically low-margin or money-losing services like primary care, behavioral health, care for stable chronic disease, and virtual care are transformed into profit centers since they generate cost savings. This turns these areas into priorities for investment in a value-based marketplace, but especially so in AMCs since they have historically been underfunded and supply-constrained. But such investments are difficult to fund since revenues are expected to fall during the transition to value-based reimbursement. Moreover, in value-based models, the flow of subsidies turns on its head, with surpluses mostly generated in primary care and transferred to support specialty and hospital services deemed to be critical. The challenge for AMCs is that value-based care models are less than a zero-sum game, since the cost savings predominantly come from the previous profit centers (specialists and hospitals) and there is less money to invest in building the services needed to lower total cost of care. The bottom line is that there is not enough money for AMCs to fully offset lost revenue from specialists and hospitalizations.^4^

If AMCs want to preserve their current operating income in value-based care, they have a few options, all of them problematic. First, they could reduce their cost structures in excess of the reduction in demand from lowering total cost of care. This kind of belt-tightening is not a core competency of most AMCs, who fear a death spiral from an exodus of currently profitable specialists if they lower salaries or support services. Moreover, the lower productivity of many AMC-based specialists is what allows such specialists to engage in research, education, and other nonclinical activities. Finally, reducing hospital size and cost structure is a contentious and complex activity, in part because every clinical service in an AMC is associated with a box on a faculty organization chart, and there are often parallel educational and research demands that are compromised by smaller services and lower funding levels.

Alternatively, AMCs may reject value-based care and try to increase margins from a falling number of high-acuity patients for which they still receive FFS reimbursement. The challenge of this alternative is that, when primary care doctors are rewarded for cutting the cost of care, they have an incentive to refer patients away from higher cost specialists and hospitals. This could lead to a much lower demand for AMCs relying on FFS and an imperative to make much higher margins on the remaining patients—rendering the organizations less and less attractive in an increasingly competitive and cost-conscious marketplace.

The challenge of transitioning to value-based care for AMCs goes beyond the redistribution of financial resources. To succeed in value-based care, new workflows are also needed. Better access to primary care is crucial so patients can be seen early in illnesses as well as after emergency department visits and hospital discharges. Periodic wellness exams are critical to delivering preventive care, coding disease acuity accurately, and achieving quality metric goals. Improving access to behavioral health is often necessary as well. Similarly, in value-based models, virtual care often emerges as a cost-effective way to check on patients efficiently.^5^ The same can be said for remote-patient monitoring and chronic care management for high-risk patients. Most AMCs lack the capacity to excel in these areas, and currently lack the motivation to invest in them.

What can AMCs do? So far, they have generally done fine by concentrating on their FFS business, and it's possible that this strategy will remain viable. But we see this as an increasingly risky bet. Instead, we are witnessing a steady movement by payors toward more value-based models. We also think that it is likely that Medicare will put its thumb on the scale and begin enacting mandatory value-based care models. If value-based models emerge as the dominant theme in our reimbursement system, AMCs will need to right-size their cost and organizational structures, with a larger outpatient focus, fewer specialists, and a compensation and promotion system that rewards more intensive primary care and other strategies that lower the total cost of care.^6^ As the share of FFS reimbursement shrinks, AMCs that are unwilling, or unable, to play well in the value market will likely focus on the highest acuity cases, essentially becoming focused factors for surgical, transplant, and intensive care unit care. This may allow them to preserve their margins and operating incomes—for a time—but will make it increasingly difficult for them to be comprehensive enough to succeed in their training and research missions.

It is premature to predict the demise of the AMC. But much of their financial success has been predicated on an FFS model whose rewards have comported with AMCs’ organizational structures and cultures. It seems likely that our reimbursement system will move to one that increasingly values prevention and efficient care. If that happens, AMCs will be forced to reinvent themselves to become institutions that not only specialize in delivering cutting edge and high-acuity care but also succeed in delivering care that is cost-effective across a range of patient populations. If they cannot do that, they are likely to become smaller components of the clinical delivery system, which will not only challenge their economic models but also have significant effects on their ability to carry out the training and research missions that our society depends on.

## Supplementary Material

qxad002_Supplementary_Data
